# Metabolomic screening of radioiodine refractory thyroid cancer patients and the underlying chemical mechanism of iodine resistance

**DOI:** 10.1038/s41598-024-61067-6

**Published:** 2024-05-08

**Authors:** Weihui Zheng, Xi Tang, Jinyun Dong, Jianguo Feng, Min Chen, Xin Zhu

**Affiliations:** 1 Key Laboratory of Head and Neck Cancer Translational Research of Zhejiang Province, Zhejiang Cancer Hospital, Hangzhou, 310022 Zhejiang China; 2 Hangzhou Institute of Medicine (HIM), Chinese Academy of Sciences, Hangzhou, 310018 Zhejiang China; 3 Institute of Research Center, Zhejiang Cancer Hospital, Hangzhou, 310022 Zhejiang China; 4 Thyroid Surgery Department, Zhejiang Cancer Hospital, Hangzhou, 310022 Zhejiang China; 5 Chongqing Institute of Advanced Pathology, Jinfeng Laboratory, Chongqing, 401329 China

**Keywords:** Radioiodine-resistant thyroid cancer, Phenylalanine, Tyrosine, Metabolic pathways, Iodination reaction, Cancer, Oncology

## Abstract

Radioiodine refractory (RAIR) patients do not benefit from iodine-131 therapy. Thus, timely identification of RAIR patients is critical for avoiding ineffective radioactive iodine therapy. In addition, determining the causes of iodine resistance will facilitate the development of novel treatment strategies. This study was comprised of 20 RAIR and 14 non-radioiodine refractory (non-RAIR) thyroid cancer patients. Liquid chromatography-mass spectrometry was used to identify differences in the serum metabolites of RAIR and non-RAIR patients. In addition, chemical assays were performed to determine the effects of the differential metabolites on iodine uptake. Metabolic pathway enrichment analysis of the differential metabolites revealed significant differences in the phenylalanine and tyrosine metabolic pathways. Notably, quinate and shikimic acid, metabolites of the tyrosine pathway, were significantly increased in the RAIR group. In contrast, the phenylalanine pathway metabolites, hippuric acid and 2-phenylacetamide, were markedly decreased in the RAIR group. Thyroid peroxidase plays an important role in catalyzing the iodination of tyrosine residues, while the ionic state of iodine promotes the iodination reaction. Quinate, shikimic acid, hippuric acid, and 2-phenylacetamide were found to be involved in the iodination of tyrosine, which is a key step in thyroid hormone synthesis. Specifically, quinate and shikimic acid were found to inhibit iodination, while hippuric acid and 2-phenylacetamide promoted iodination. Abnormalities in phenylalanine and tyrosine metabolic pathways are closely associated with iodine resistance. Tyrosine is required for thyroid hormone synthesis and could be a potential cause of iodine resistance.

## Introduction

Thyroid cancer is the most common endocrine tumor. Since 2013, the incidence of differentiated thyroid cancer has increased annually at a rate of 3% per year^1^ and the annual incidence rate of papillary thyroid cancer larger than 2 cm has continued to rise^2^. Most patients with differentiated thyroid cancer, especially those with papillary thyroid cancer, have a good prognosis with the 10-year survival rate of patients undergoing surgery and iodine-131 treatment greater than 90%^3^. However, approximately 5% of patients develop resistance to radioiodine therapy. In these cases, patients are unable to take up radioactive iodine and show a significantly worse prognosis with a 10-year survival rate of less than 10%. Locally advanced progressive thyroid cancer with iodine resistance is the leading cause of thyroid cancer mortality^4–6^.

Iodine resistance, defined as no iodine uptake or the gradual progression from iodine uptake to no iodine uptake, occurs during the initial iodine-131 treatment^7^. Iodine-resistant thyroid cancer is a challenging issue in the clinical diagnosis and treatment of thyroid cancer. Patients with iodine-resistant thyroid cancer are usually treated with repeated or increased doses of iodine-131. However, the treatment is usually unsuccessful and very likely to cause systemic radiation side effects associated with cumulative doses, such as radiation pneumonia or pulmonary fibrosis, salivary gland damage, and bone marrow suppression. Patients with iodine resistance are often prone to locally advanced thyroid cancer or distant metastasis. Thus, timely identification of iodine-resistant tumors would be beneficial for providing patients with the most effective therapeutic strategies.

Metabolomics is a high-throughput analytical technique that provides a comprehensive and systematic description of the metabolites (small molecules below 1500 Da) present in biological samples^8,9^. Metabolites are by-products of functional pathways, and their profiles can accurately reflect changes in human vital functions, providing valuable information on health and disease conditions. In particular, the metabolism of amino acids plays an important role in the metabolism, growth, maintenance, and repair of tissues^10,11^.

**Figure 1:**
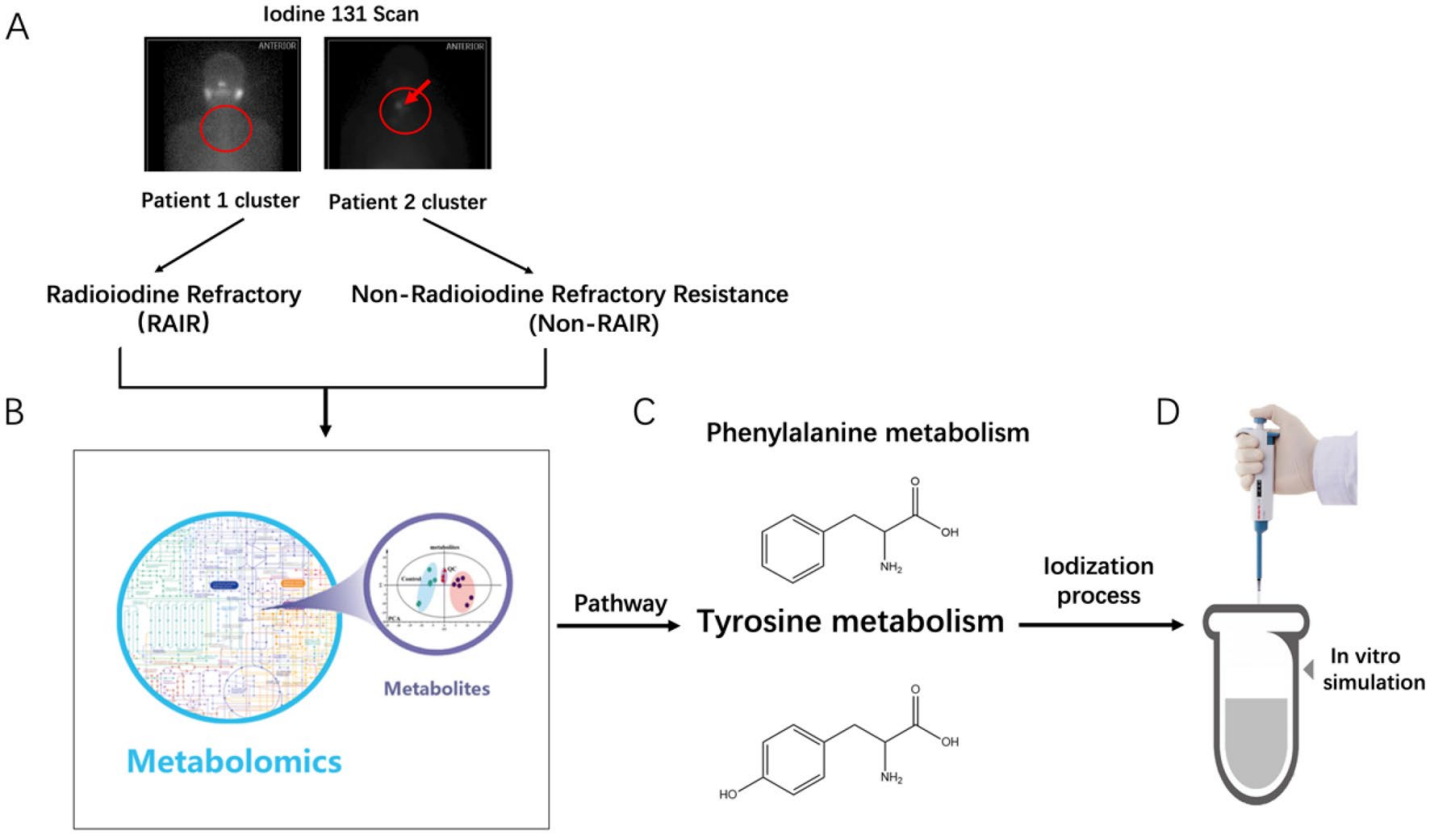
Research schematic diagram. (**A**) Patients were divided into radioiodine resistance and non-radioiodine resistance groups, based on their clinical and imaging data. (**B**) Metabolomic detection and analysis. (**C**) Two metabolic pathways, phenylalanine metabolism and tyrosine metabolism, were found to be abnormal. (**D**) Chemical simulation of the tyrosine iodination reaction in vitro.

In recent years, metabolomics has received extensive attention and has been used for the early diagnosis of many types of cancer including thyroid cancer. Studies have shown that there are significant differences between thyroid tumor tissues and normal counterparts, while amino acids were found to display distinct expression profiles in thyroid cancer tissues of different pathological types^11–13^. To date, little is known about whether metabolites play an important role in radioiodine refractory (RAIR) thyroid cancer. Moreover, there have been no reports on the application of metabolomics to screen iodine-resistant patients. Therefore, we conducted a non-targeted tissue metabolomics study on RAIR and non-RAIR thyroid cancer patients using liquid chromatography-mass spectrometry (LC–MS) to identify metabolic differences in patients who might be the basis for the early diagnosis of iodine resistance in thyroid cancer. In addition, this study explored the potential chemical mechanisms underlying iodine resistance based on the results of metabolomics in combination with the metabolic pathway of iodine. The outline of our study is shown in Fig. 1. To the best of our knowledge, the present study is the first to apply metabolomics to identify iodine resistance in thyroid cancer patients.

## Materials and methods

### Clinical data of patients undergoing iodine-131 therapy

Thirty-four thyroid cancer patients who underwent iodine-131 therapy after total resection of the cancer in Zhejiang Cancer Hospital (Hangzhou, China) between January 2018 and December 2021 were enrolled in this study and grouped according to the diagnostic criteria for RAIR and non-RAIR thyroid cancer outlined in the 2015 American Thyroid Association management guidelines. All 20 cases in the RAIR group met the following criteria: (1) iodine-131 was not concentrated on malignant/metastatic tissue outside the thyroid bed at the time of the initial therapeutic whole-body scan; (2) iodine-131 uptake was evident but did not accumulate later in the tumor tissue; (3) iodine-131 uptake was only retained in certain lesions; and (4) metastatic disease progressed despite the presence of massive iodine-131 uptake. The remaining 14 cases did not fall into any of the above four categories and were classified as non-RAIR thyroid cancer.

### Methodology and analysis of LC–MC-based metabolomics

The metabolomics analysis workflow consisted of sample collection (Whole blood samples were obtained from patients before their first surgery), sample quality control, experimental testing, experimental reporting, and bioinformatics data analysis. More in-depth details about these methods can be found in the supplementary material.

### Relationship between clinicopathological factors and abnormal metabolic products

The relationship between the differential metabolites identified by metabolic screening and eight clinicopathological characteristics, including gender, age, number of tumors, capsule invasion, extra-glandular invasion, lymph node metastasis, number of lymph node metastasis, and tumor, node, and metastasis (TNM) staging of the tumor, were examined.

### The chemical mechanism of iodine resistance for TPO

The chemical mechanism of iodine resistance was examined using an in vitro iodination reaction model that simulated iodination at the apical membrane surface of thyroid cells. Dulbecco’s phosphate buffered saline (DPBS, pH 7.4) was used to simulate the matrix for the iodination reaction in thyroid cells. The following six different chemical reaction systems were established: I, tyrosine (Tyr), iodine anion (I^−^), hydrogen peroxide (H_2_O_2_), and tyrosine peroxidase (TPO); II, Tyr, iodine (I_2_), H_2_O_2_, and TPO; III, Tyr, I^−^/I_2_, H_2_O_2_, and TPO; IV, Tyr, I_2_, and TPO; V, Tyr and I_2_; and VI, Tyr and I^−^. Chemical reactions were carried out for 24 h at 37 °C, then characterized by LC–MS and infrared analyses to investigate the factors influencing the iodination reaction, as well as the effects of compounds identified by metabolomics screening.

### Statistical analysis

Statistical analysis was performed using Prism software (La Jolla, CA, USA) for data of clinical characteristics, and continuous data were analyzed using the unpaired *t*-test. Count data were analyzed using the Chi-squared test and Fisher's exact test. A two-sided p value < 0.05 was considered statistically significant. Statistical analysis for the metabolomics approaches, mainly including Principal component analysis (PCA), heat map analysis, ROC curve analysis, and metabolic pathway analysis using the metaboanalyst database (http://www.Metaboanalyst.ca).

### Informed consent

Informed consent was obtained from all individuals included in this study. The images of participate patients have been allowed to publish.

### Ethical approval

The study protocol was prepared according to the Helsinki Declaration and approved by the Medical Ethics Committee of Zhejiang Cancer Hospital (Decision No: IRB-2020-438 Ke). Patients have signed informed consent regarding publishing their data and image pictures.

## Results

### Patient information

Twenty RAIR and 14 non-RAIR thyroid cancer patients who were randomly admitted into the hospital between January 2018 and December 2021 were enrolled in this study. The clinicopathological characteristics of the enrolled patients including age, gender, number of tumors, capsule invasion, extra-glandular invasion, lymph node status, and TNM staging are shown in Table 1. The Chi-square test indicated that there were no statistically significant differences in the clinicopathological characteristics between the RAIR and non-RAIR groups (p-value > 0.05) (Table 1).

**Table 1:** Clinical characteristics of the participants.

	Non-RAIR group	RAIR group	*P* value
	(n = 14)	(n = 20)	
Sex			0.868
Male	6	8	
Female	8	12	
Age			0.726
< 55 y	9	14	
≥ 55 y	5	6	
Number of tumors			0.999
1	4	6	
≥ 2	10	14	
Capsule invasion			0.999
Yes	13	18	
No	1	2	
Extra-glandular invasion			0.999
Yes	10	15	
No	4	5	
Lymph-node metastasis			0.283
Yes	11	19	
No	3	1	
Number of lymph node metastasis			0.228
< 5	5	3	
≥ 5	9	17	
TNM stage			0.458
I–II stage	8	15	
III–IV stage	6	5	

**Figure 2:**
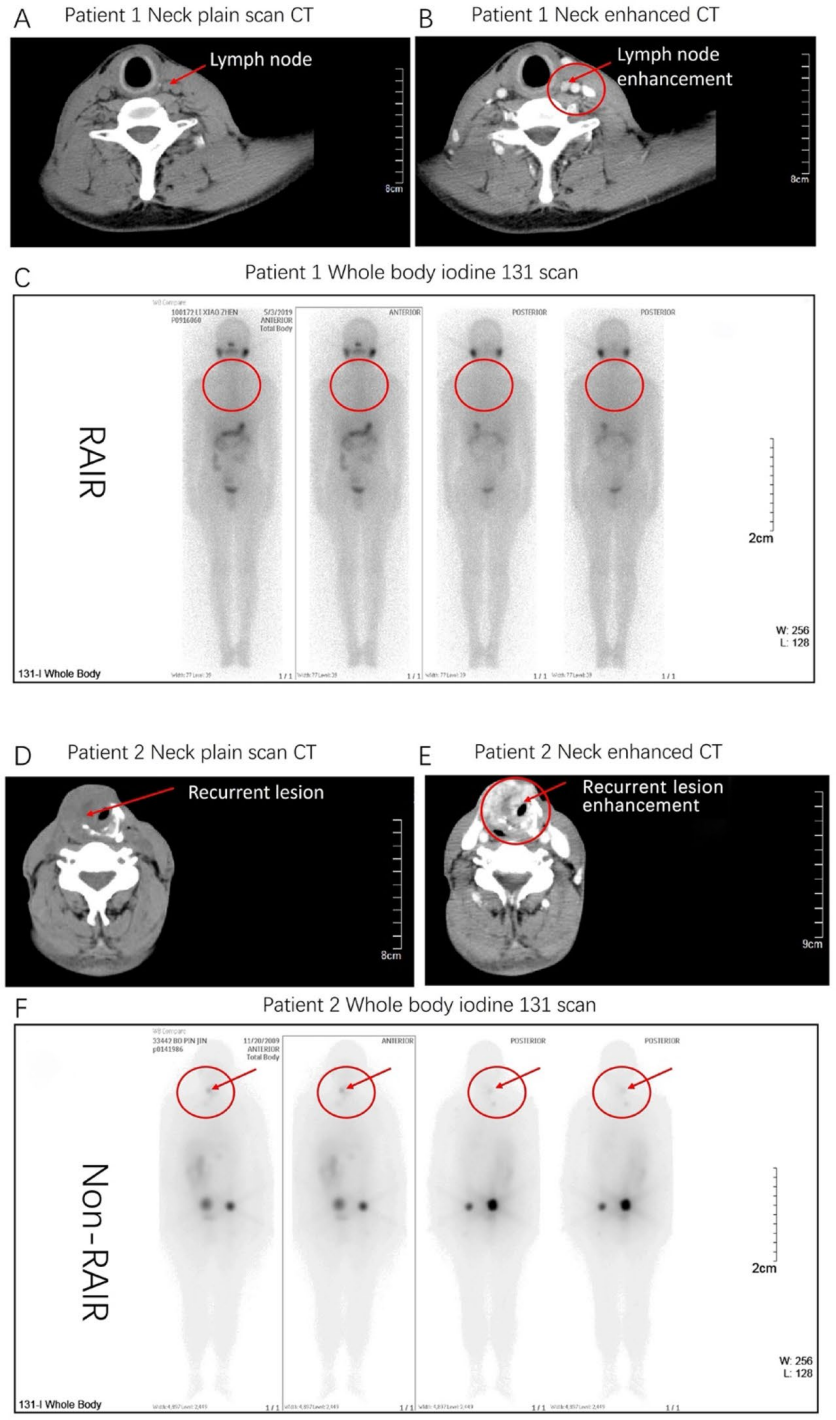
Typical imaging of patients with radioiodine resistance (RAIR) and non-radioiodine resistance (non-RAIR). (**A**) Plain CT scan of the neck of an RAIR patient showing the left cervical carotid sheath, and lymph nodes approximately 1 cm in size (red arrow). (**B**) Enhanced CT scan of the neck of an RAIR patient showing significant enhancement of the lymph nodes (red arrow). (**C**) Whole body iodine-131 scan of RAIR patients showing no foci of iodine uptake in the neck lymph nodes (red circle area). (**D**) CT scan of the neck of a non-RAIR patient showing a recurrent lesion of the pretracheal thyroid approximately 4 cm in size (red arrow). (**E**) Enhanced CT scan of the neck of a non-RAIR patient showing obvious enhancement and necrosis (red arrow). (**F**) Whole body iodine-131 scan of non-RAIR patients showing iodine absorption lesions in the red circle of neck (red arrow).

Typical imaging manifestations of RAIR and non-RAIR thyroid cancer are shown in Fig. 2. Figure 2A–C show a case presenting with enhancement of the neck lymph nodes by CT scan, confirmed malignancy by aspiration biopsy, and the absence of iodine uptake in the neck in the whole-body iodine-131 scan. A diagnosis of RAIR thyroid cancer was considered. Figure 2D–F present a case of recurrent pre-tracheal lesions. In this case, the CT scan revealed significant enhancement, while the whole-body iodine-131 scan indicated that iodine uptake had occurred in the neck lesions. Hence, the patient was diagnosed with non-RAIR thyroid cancer.

### Metabolomics analysis

#### Metabolic profiling

We selected three shared chromatographic peaks in the positive ion mode and two shared chromatographic peaks in the negative ion mode and examined the stability of retention time and peak area in the RAIR and non-RAIR groups. The relative standard deviation (RSD) of the retention time of the chromatographic peaks and RSD of the peak area in the positive and negative ion modes were calculated. We found that the proportion of characteristic peaks with an RSD < 30% exceeded 70%, indicating that the quality control of the data was good, and that the analysis system was robust and reliable (Fig. S1).

#### Multivariate statistical analyses

**Figure 3:**
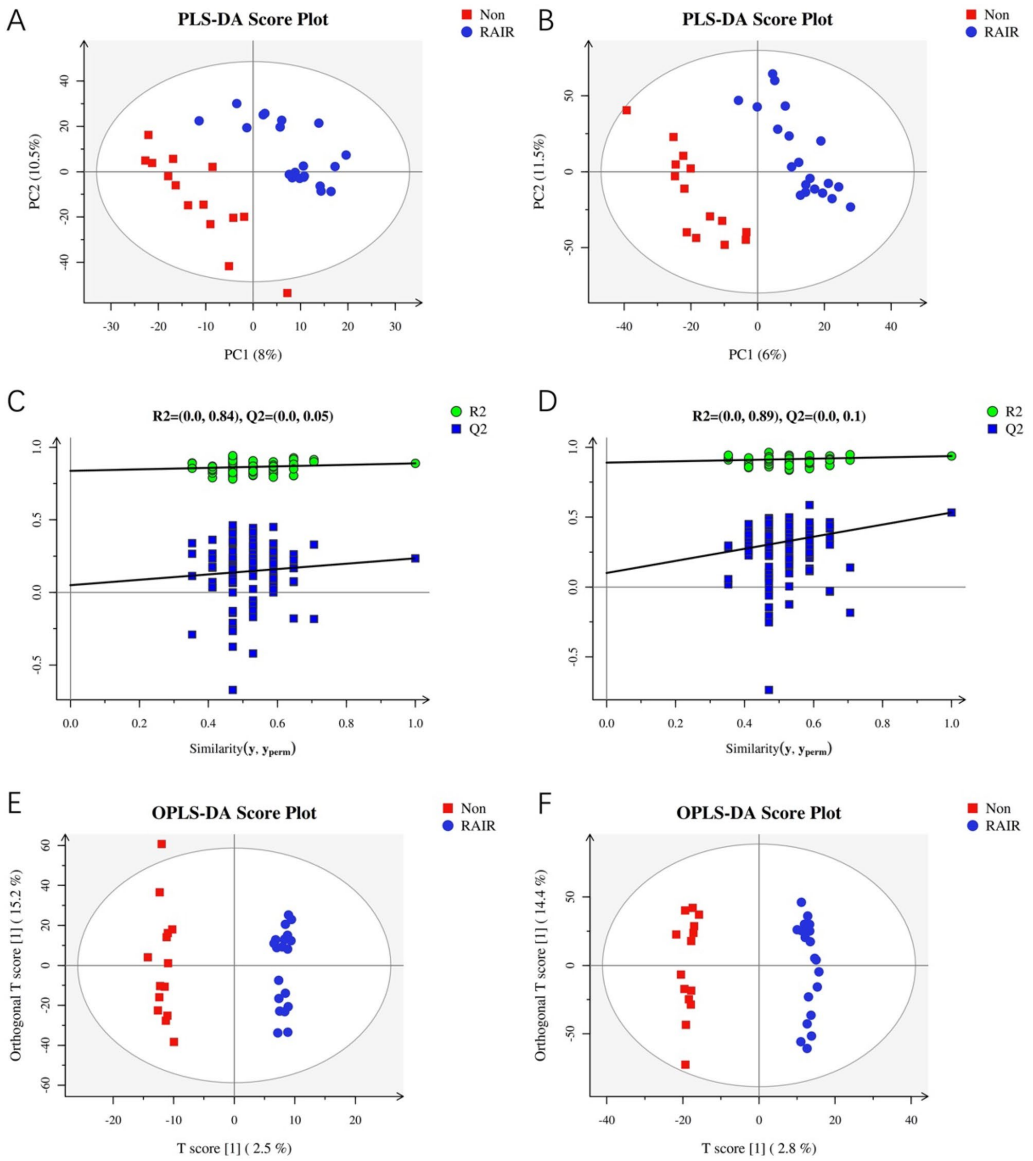
Score plots and permutations for multivariate statistical analysis. (**A**) PLS-DA score plot for positive mode. (**B**) PLS-DA score plot for negative mode. (**C**) PLS-DA permutation plot for positive mode. (**D**) PLS-DA permutation plot for negative mode. (**E**) OPLS-DA score plot for positive mode. (**F**) OPLS-DA score plot for negative mode. VIP from Orthogonal Projections to Latent Structures Discriminant Analysis (OPLS-DA).

After data processing, 5462 and 10,965 precursor molecules were obtained in the positive and negative ion modes, respectively. The two data sets were analyzed using partial least squares-discriminant analysis (PLS-DA). As shown in Fig. 3A,B, the models had good goodness of fit and predictive power (positive ion mode: R^2^Y = 0.889, Q^2^ = 0.235; negative ion mode: R^2^Y = 0.937, Q^2^ = 0.533). The permutation test was then performed to determine whether the current PLS-DA model was overfitting. As depicted in Fig. 3C,D, the slope of the Q2 regression line of the permutation test was greater than 0, indicating that the model was not overfitting. Finally, supervised orthogonal-partial least squares discriminant analysis (OPLS-DA) revealed that the RAIR and non-RAIR groups were distinguishable in the positive and negative ion modes (Fig. 3E,F), while the established models had good predictive ability (positive ion mode: R2Y = 0.991; negative ion mode: R2Y = 0.991). In addition, the VIP values reflected the importance of each variable in model building. In this study, VIP ≥ 1 and one-way ANOVA (p ≤ 0.05) were used as cut-off criteria.

#### Metabolite screening and metabolic pathway analysis

**Figure 4:**
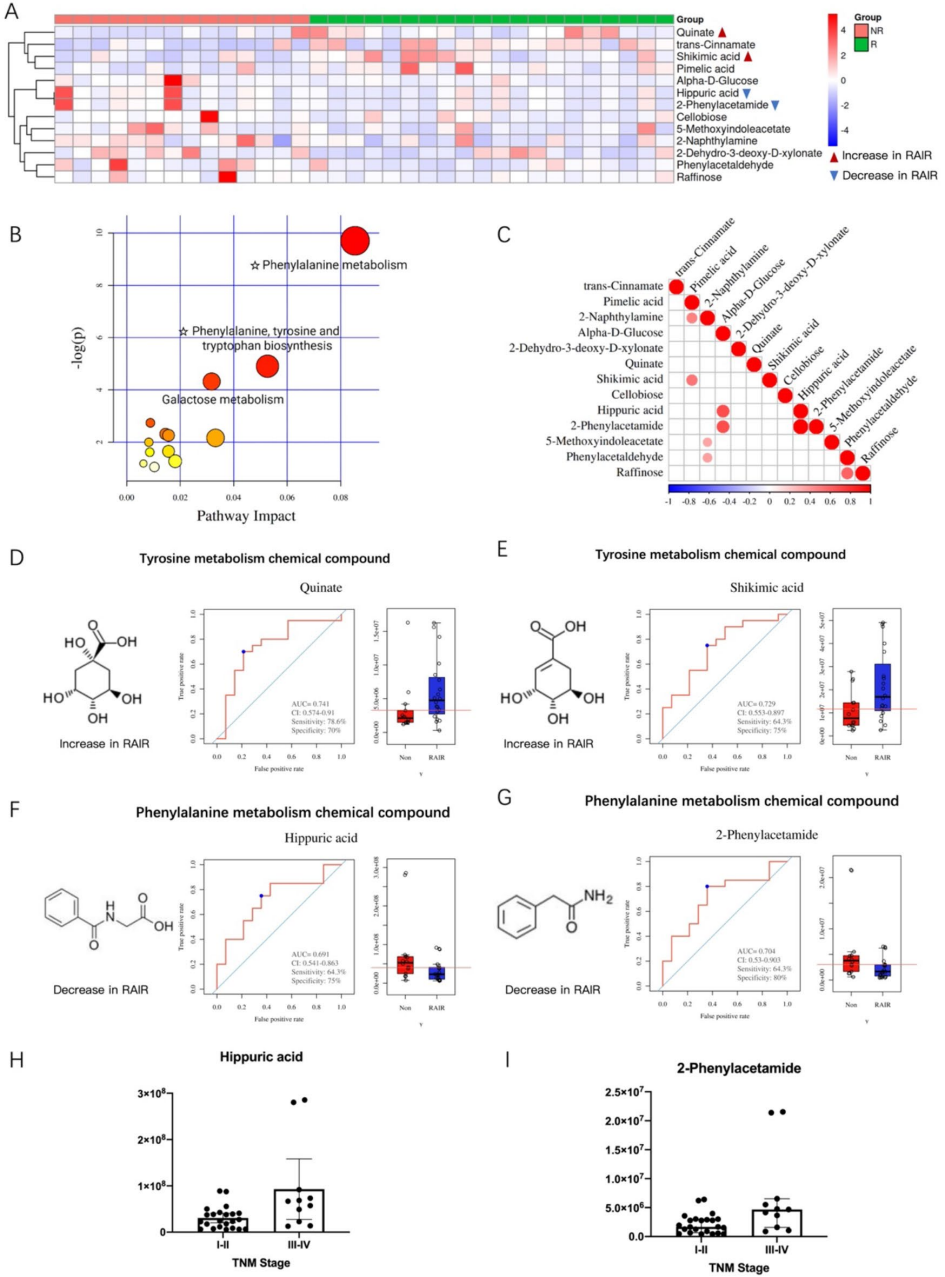
The main metabolic pathways identified by the screening of differential compounds are the phenylalanine and tyrosine metabolic pathways. (**A**) Heat map of differential metabolites. (**B**) Summary of pathway analysis using MetaboAnalyst. (**C**) Heat map of differential metabolite associations. (**D**) Increased expression of quinate in the metabolic tyrosine pathway in RAIR patients and its receiver operating characteristic (ROC) curve. (**E**) Increased expression of shikimic acid in the tyrosine metabolic pathway in RAIR patients and its ROC curve. (**F**) Decreased expression of hippuric acid in the phenylalanine metabolic pathway in RAIR patients and its ROC curve. (**G**) Decreased expression of 2-phenylacetamide in the phenylalanine metabolic pathway in RAIR patients and its ROC curve. (**H**) Hippuric acid is associated with tumor, node, and metastasis (TNM) staging of thyroid cancer. (**I**) 2-Phenylacetamide is associated with TNM staging of thyroid cancer.

Comparative analysis led to the identification of 13 differential metabolites between the RAIR and non-RAIR groups (Table 2), which were then subjected to heat map analysis (Fig. 4A). In addition, the MetaboAnalyst database (http://www.metaboanalyst.ca) was used to analyze the metabolic pathway data. As shown in Fig. 4B, the phenylalanine and tyrosine metabolic pathways were found to be the most significantly different between the RAIR and non-RAIR groups. Figure 4C shows the correlation analysis. Among the 13 metabolites, quinate and shikimic acid in the phenylalanine metabolic pathway were elevated in the RAIR group (Fig. 4D,E). In contrast, hippuric acid and 2-phenylacetamide in the tyrosine metabolic pathway were reduced in the RAIR group (Fig. 4F,G). Furthermore, hippuric acid and 2-phenylacetamide were significantly correlated with the TNM staging of thyroid cancer (Fig. 4H,I). The ROC curves for the remaining nine differential metabolites are shown in Fig. S2.

**Table 2:** Identification of significant differential metabolites.

No.	Name	Formula	m/z	rt	VIP	p value	ppm
1	Quinate^○^	C_7_H_12_O_6_	173.0430	207.6955	1.7690	0.0165	11.8146
2	Shikimic acid^○^	C_7_H_10_O_5_	173.0443	174.5205	2.2972	0.0263	7.0090
3	Hippuric acid^△^	C_9_H_9_NO_3_	178.0497	333.3320	2.1220	0.0342	2.6307
4	2-Phenylacetamide^△^	C_8_H_9_NO	134.0610	333.3320	2.1109	0.0373	0.9805
5	Trans-Cinnamate	C_9_H_8_O_2_	131.0491	565.0450	3.5324	0.0014	0.3421
6	Pimelic acid	C_7_H_12_O_4_	160.0758	65.4221	1.9611	0.0150	13.6694
7	Phenylacetaldehyde	C_8_H_8_O	119.0503	515.5830	2.0338	0.0480	0.3321
8	5-Methoxyindoleacetate	C_11_H_11_NO_3_	204.0664	443.3430	1.6068	0.0373	0.8294
9	2-Naphthylamine	C_10_H_9_N	143.0730	392.9780	1.9223	0.0314	3.6618
10	Alpha-d-glucose	C_6_H_12_O_6_	161.0443	259.1190	1.8200	0.0074	4.1731
11	2-Dehydro-3-deoxy-d-xylonate	C_5_H_8_O_5_	147.0304	284.5930	1.8781	0.0150	3.3239
12	Cellobiose	C_12_H_22_O_11_	341.1074	95.5473	1.3102	0.0287	1.7530
13	Raffinose	C_18_H_32_O_16_	485.1650	380.8000	1.7705	0.0480	29.5822

^○^Metabolites with abnormal phenylalanine metabolic pathway. ^△^Metabolites with abnormal tyrosine metabolic pathway. VIP ≥ 1 and single factor analysis of variance (ANOVA) were used as truncation criteria.

### The chemical mechanism of iodine resistance

#### Identification of key factors influencing the iodination reaction

The chemical reaction formula for the conversion of phenylalanine to tyrosine and the reaction of tyrosine with iodine to generate thyroid hormone constitutes the theoretical basis for studying tyrosine iodination in the current study (Fig. S3).

**Figure 5:**
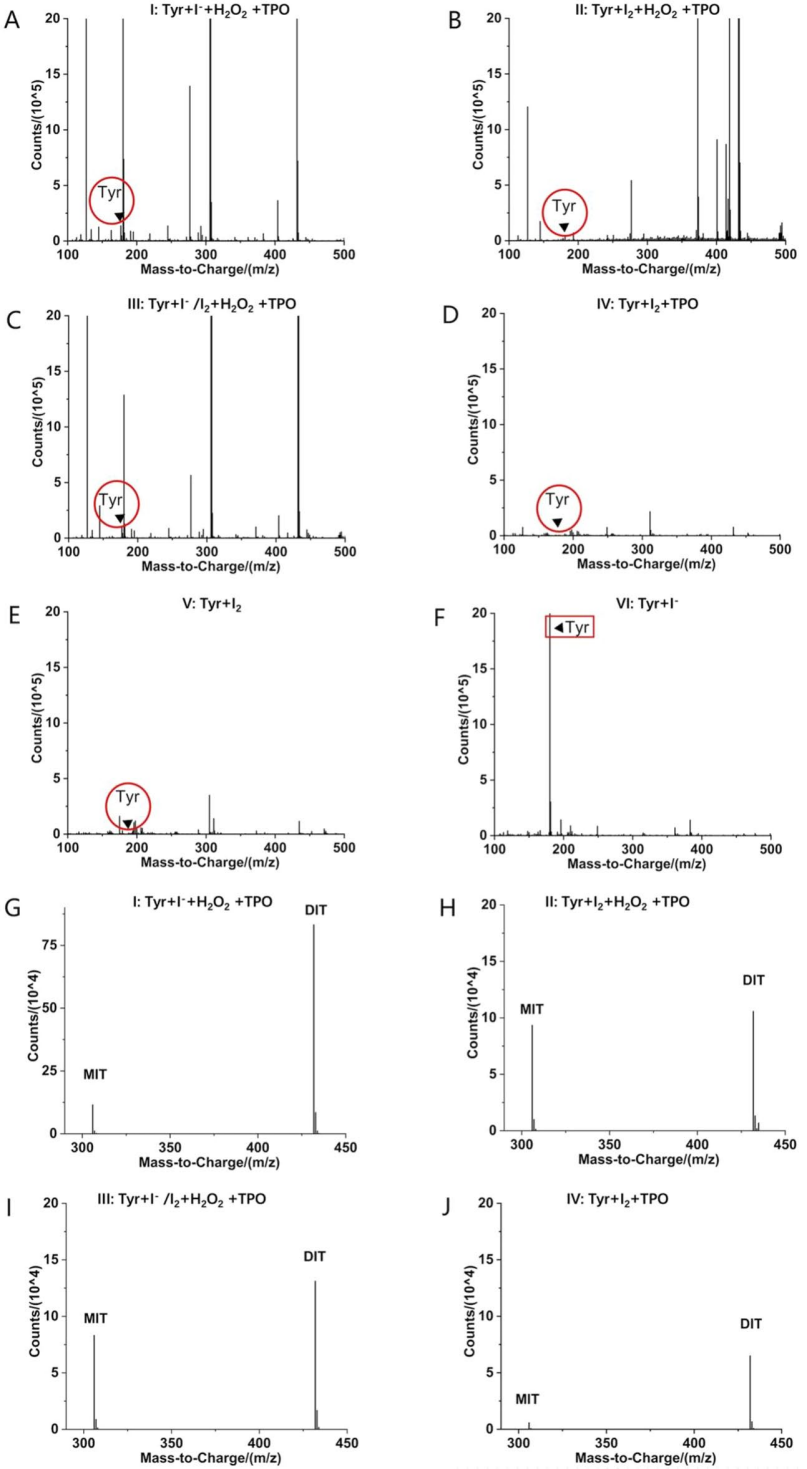
LC–MS characterization of full spectrum and identification spectrum of MIT and DIT. (**A**) Tyr is low in system I (Tyr + I^−^ + H_2_O_2_ + TPO). (**B**) Tyr is low in system II (Tyr + I_2_ + H_2_O_2_ + TPO). (**C**) Tyr is low in system III (Tyr + I^−^/I_2_ + H_2_O_2_ + TPO). (**D**) Tyr is low in system IV (Tyr + I^2^ + TPO). (**E**) Tyr is low in system V(Tyr + I_2_). (**F**) Tyr is high in system VI (Tyr + I^−^). MIT and DIT are produced in systems I, II, III, and IV (**G**–**J**) but not in systems V and VI (no curve).

Figure 5 shows the mass spectra of the six chemical reaction systems after incubation for 24 h (A, I; B, II; C, III; D, IV; E, V; and F, VI). We found that the tyrosine peaks were greatly reduced in systems I–V compared to system VI. These findings suggested that tyrosine was not significantly involved in the chemical reaction occurring in system VI in the presence of I^−^ only. Mass spectrometry analysis revealed that both mono-iodotyrosine (MIT) and di-iodotyrosine (DIT) were not produced in the absence of TPO (system V). MIT and DIT peaks were observed in the presence of TPO (system IV), while the addition of H_2_O_2_ (system II) resulted in increased levels of MIT and DIT. Finally, significantly higher levels of MIT and DIT were observed in system I in which I^−^ was initially added than the other systems. Our data indicate that TPO plays a key role in tyrosine iodination, while H_2_O_2_ promotes iodination further. Our data also show that tyrosine iodination is facilitated when iodine is initially in its anionic form (I^−^) (Table S1).

#### Effects of tyrosine metabolites produced during the iodination reaction

**Figure 6:**
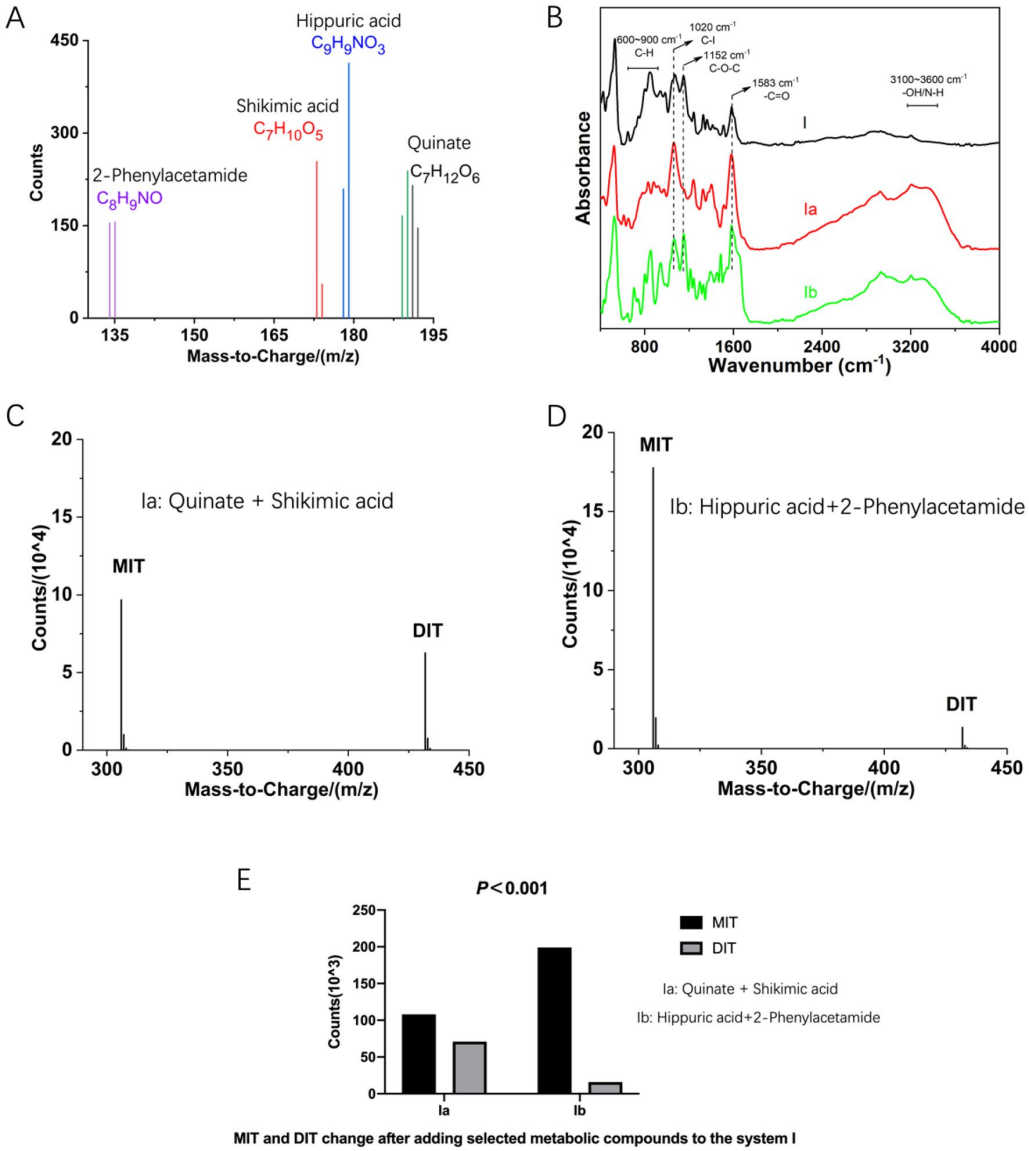
LC–MS and infrared characterization of the iodination reaction in system I. (**A**) LC–MS identification spectra of quinine, shikimic acid, 2-phenylacetamide and hippuric acid in system I. (**B**) a (quinate, shikimic acid) and b (2-phenylacetamide, hippuric acid) were introduced into the infrared characterization of system I. (**C**,**D**) Identification spectra of MIT and DIT for systems Ia and Ib. (**E**) The abundance of MIT and DIT in system I was analyzed following addition of the small organic molecules a and b.

As shown in Fig. 6A,B, tyrosine was involved in the iodination reaction in the presence of I^−^, which produced small amounts of quinate (C_7_H_12_O_6_), shikimic acid (C_7_H_10_O_5_), hippuric acid (C_9_H_9_NO_3_), and 2-phenylacetamide (C_8_H_9_NO) in the presence of TPO or H_2_O_2_.

The production of these organic small molecule metabolites is thought to occur during tyrosine iodination in the human body. Over time, the levels of some of these metabolites are gradually reduced following metabolism in the kidneys via the blood circulation. In addition, some metabolites may accumulate in the body to a certain extent due to various reasons. Here, we performed in vitro chemical simulation of the iodine conversion reaction to investigate the effects of these small molecule metabolites accumulated in vivo on iodine conversion trends. In our experiments, quinate and shikimic acid were assigned to Group a, while 2-phenylacetamide and hippuric acid were assigned to Group b. System I was selected for subsequent studies.

The small organic molecules of Group a and Group b were added to system I to form system Ia and system Ib, respectively. Systems Ia and Ib were incubated for 24 h at 37 °C, then characterized by the infrared spectra (Fig. 6B). System I displayed a higher absorption peak intensity at long wavelengths, indicating that DIT and tetra-iodothyroxine (T_4_) were enriched. In particular, this system showed a strong characteristic absorption peak at 1152 cm^−1^, which was mainly derived from the C–O–C stretching vibration, suggesting that the reaction products of the system contained relatively high levels of tri-iodothyroxine (T_3_) or T_4_. System Ia exhibited enhanced hydroxyl and N–H stretching vibrations and the shift of the absorption peak towards long wavelengths in the 3100–3600 cm^−1^ range, indicating that the number of hydroxyl structures had increased in system Ia (Fig. 6B). Moreover, the absorption peaks in the 700–1000 cm^−1^ range were significantly red-shifted while displaying a jagged fluctuation, representing the presence of various aromatic hydrocarbon substitutions, as well as both MIT and DIT. Furthermore, disappearance of the characteristic C–O–C peak (1152 cm^−1^) indicated the almost complete absence of T_3_ and T_4_ type intermediates. Compared with system I, system Ia showed a decrease in iodinated products, which was consistent with the LC–MS data (Figs. 3 and 6C). These findings indicated that both quinate and shikimic acid had an inhibitory effect on system I.

System Ib showed a similar change in the hydroxyl band as system Ia, indicating that the free hydroxyl structures in the system had increased, mainly due to the addition of small molecules. Notably, two strong characteristic absorption peaks at 1020 cm^−1^ and 1152 cm^−1^ were observed, which were mainly derived from the stretching vibrations of C–I and C–O–C, respectively. This observation indicated that the reaction products contained relatively high levels of MIT, DIT, T_3_, and T_4_ (Fig. 6B, green line). As shown in Fig. 6D,E, the abundance of MIT was significantly increased, and the level of DIT was decreased in system Ib compared with system I. Thus, combined with the infrared spectra, we concluded that system Ib resulted in an increase in T_3_ and T_4_. These results indicated that the addition of group Ib small molecules to system I promoted iodination.

Overall, this study found that quinate and shikimic acid inhibited iodination, whereas 2-phenylacetamide and hippuric acid promoted iodination.

## Discussion

### The main findings of this study

Here, we performed metabolomics analysis to identify differential metabolites in the serum of iodine-resistant and non-iodine-resistant thyroid cancer patients. We found that in the iodine-resistant patients, quinate and shikimic acid were significantly increased, while hippuric acid and 2-phenylacetamide were markedly decreased. Strikingly, the differential metabolites were mainly enriched on the phenylalanine or tyrosine metabolic pathways. Phenylalanine can be converted to tyrosine. Chemical analysis revealed that quinate, shikimic acid, hippuric acid, and 2-phenylacetamide were associated with the iodination of tyrosine during thyroid hormone biosynthesis. Therefore, abnormal levels of components of the phenylalanine and tyrosine metabolic pathways may serve as markers for patients with iodine-resistant thyroid cancer. Given that tyrosine is closely associated with thyroid hormone synthesis, these findings may help unveil the potential cause of iodine resistance and show a possible therapeutic value.

### Relationship between the phenylalanine metabolic pathway and thyroid cancer

Phenylalanine is an essential amino acid that cannot be synthesized by the human body and must therefore be obtained from the diet. Phenylalanine is essential for protein synthesis, as well as the synthesis of phenylalanine and its derivatives, such as dopamine, norepinephrine, and melanin.

Studies have shown that the metabolites of the phenylalanine metabolic pathway differ significantly between the serum and tissues of thyroid cancer patients. Zhao et al. analyzed the serum metabolic profiles of patients with papillary thyroid cancer and healthy subjects using nuclear magnetic resonance (NMR) spectroscopy and found differences in the tyrosine and phenylalanine metabolic pathways between the patients and healthy individuals^12^.

Several histological studies examining the metabolic profile of tissues using high-resolution rotational NMR have shown that the thyroid tissue in papillary thyroid carcinoma (PTC) patients contains higher levels of phenylalanine and tyrosine than normal counterparts^14–16^. In 2018, Rezig et al. used metabolomics to examine thyroid follicular adenoma tissue and thyroid cancer tissue and found that malignant tumors were positively correlated with the levels of amino acids, such as tyrosine, serine, alanine, leucine, and phenylalanine^17^.

### Relationship between the tyrosine metabolic pathway and thyroid cancer

Tyrosine is a non-essential amino acid that can be synthesized from phenylalanine. Tyrosine plays an important role in the production of proteins that are components of signal transduction pathways, by acting as the recipient of phosphate groups transferred via tyrosine kinases. In turn, tyrosine kinases have been implicated in the regulation of cell proliferation, survival, differentiation, function, and motility, and are therefore associated with various cancer phenotypes^11^.

The pathway that mediates the conversion of phenylalanine to tyrosine deserves our attention because tyrosine is a vital amino acid involved in the synthesis of thyroid hormones. Thyroid hormones secreted by the thyroid gland are iodine-containing amino acid derivatives. Among them, DIT is a thyroid hormone analog, as well as an intermediate of liothyronine (tri-iodothyronine, iodothyronine). Therefore, from a chemical point of view, phenylalanine metabolism is closely associated with thyroid cancer.

Herein, we performed statistical analysis of 13 differential metabolites identified in this study and the clinical parameters of patients with thyroid cancer. We found that hippuric acid and 2-phenylacetamide in the tyrosine metabolic pathway were significantly associated with the TNM staging of the patients. Moreover, we showed that the serum levels of hippuric acid and 2-phenylacetamide in stage III–IV patients were significantly higher than those in stage I–II patients. Given that TNM staging is an important indicator of malignancy in PTC, our results suggest that tyrosine metabolism plays an important role in the occurrence and development of thyroid cancer.

### The chemical mechanism of iodination

TPO, an enzyme produced by thyroid follicular cells, is important for the synthesis of thyroid hormones. TPO catalyzes the iodination reaction between tyrosine and active iodine on the luminal surface of the microvilli of thyroid epithelial cells to produce iodotyrosines (MIT and DIT). TPO then catalyzes the subsequent coupling reaction between two iodotyrosines to produce the thyroid hormones, T_3_ and T_4_^18–20^. In this study, we showed that some tyrosine residues underwent iodination in the presence of TPO, while in the absence of TPO virtually no iodinated tyrosine was produced. Although tyrosine has a phenolic hydroxyl structure and is prone to oxidation in the presence of oxides, a higher concentration of I_2_ was found to have stronger oxidative properties in aqueous solution^21^. In system IV, tyrosine was found to be involved in two types of competing reactions: iodination and oxidation. Thus, TPO plays a highly efficient catalytic role in the iodination of tyrosine, as well as an important role in the oxidation reaction^22–24^. In systems II and IV, we found that dilute H_2_O_2_ possessed weak oxidizing properties and exerted an activating effect on the ortho structure of the tyrosine benzene ring, thereby facilitating the ortho iodination of tyrosine^25^. System I, which contained I^−^ in the presence of TPO, H_2_O_2_ and tyrosine displayed high efficiency iodination of tyrosine In this case, I^−^ was converted individually into low concentration and high activity I_2_ in the presence of TPO and H_2_O_2_. Following activation of the benzene ring of tyrosine by H_2_O_2_, the low concentration of I_2_ can effectively participate in the ortho iodination reaction, while barely participating in the competing oxidation reaction.

Quinate, shikimic acid, 2-phenylacetamide, and hippuric acid have been shown to be produced during the iodination of tyrosine. Metabolomic analysis has shown that these organic small molecules are enriched in human blood. Furthermore, a higher abundance of quinate and shikimic acid was detected in the serum of iodine-resistant patients, while the serum levels of 2-phenylacetamide and hippuric acid were significantly reduced in patients with better iodine uptake. Thus, we proposed that quinate, shikimic acid, 2-phenylacetamide, and hippuric acid may affect iodine uptake by the thyroid gland and sought to determine whether this was the case in the current study. We found that introduction of a large amount of quinate and shikimic acid into system I led to inhibition of iodination, while addition of a large amount of hippuric acid and 2-phenylacetamide promoted iodination.

### The effects of different numbers of molecules on the benzene ring of phenylalanine and tyrosine metabolites

In the present study, four metabolites were identified by screening the phenylalanine and tyrosine pathways. We further examined the number of molecules on the benzene ring of the identified compounds and found that the phenylalanine metabolites quinate and shikimic acid had at least three hydroxyl molecules attached to the benzene ring, while the tyrosine metabolites 2-phenylacetamide and hippuric acid had no hydroxyl groups or other groups attached to the benzene ring. These findings may provide the chemical basis for attaching iodine to the benzene ring. Since phenylalanine can be converted into tyrosine in the human body, and since the iodination of tyrosine is an early reaction during thyroid hormone synthesis, we hypothesized that the phenylalanine and tyrosine metabolic pathways may affect thyroid hormone synthesis, as well as iodine resistance in thyroid cancer.

There are several limitations to our study. First, it is possible that metabolites identified in the serum might not be a true representation of what is happening in the thyroid or other areas of the human body. However, since peripheral blood samples are easy to obtain, the present study may provide a reference point for further studies and non-invasive early diagnosis in the future. Second, the mechanism at the cellular or organismal levels has yet to be explored. However, our findings at the chemical level may facilitate further research at the biological level. Finally, the number of patients enrolled in this study was small, due to difficulties in obtaining samples from patients undergoing iodine therapy. However, we identified differences in metabolites between the two groups of patients based on the limited number of samples, suggesting an important role for the associated metabolic pathways. Our findings may therefore be beneficial for addressing clinical issues.

In conclusion, the present study revealed differences in the metabolites of the phenylalanine and tyrosine metabolic pathways between iodine-resistant thyroid cancer patients and non-iodine-resistant counterparts based on metabolomics analysis. Our findings may, therefore, further the understanding of iodine resistance from a chemical perspective. Exogenous supplementation of hippuric acid and 2-phenylacetamide of the tyrosine metabolic pathway may potentially improve the iodine resistance status, providing a novel approach for improving the therapeutic efficacy of iodine resistance.

### Supplementary Information

Supplementary Information.

## Data Availability

The datasets generated during and/or analyzed during the current study are available in the [Metabolism] repository, [https://pan.baidu.com/s/168SlLuaqpXms3oVhWG7SKw?pwd=9fhp]. The datasets generated during and/or analyzed during the current study are available from the corresponding author on reasonable request. All data generated or analyzed during this study are included in this published article and its supplementary information files.
