# Supplementary material for: Metabolomic screening of radioiodine refractory thyroid cancer patients and the underlying chemical mechanism of iodine resistance
Source: Sci Rep. 2024 May 8;14:10546. doi: 10.1038/s41598-024-61067-6 (PMC11079026; doi:10.1038/s41598-024-61067-6)
Supplement: Supplementary file 1 — Supplementary Information. [file 41598_2024_61067_MOESM1_ESM.docx]

Supplementary data

Metabolomics approaches

**1 Sample preparation**

Whole blood samples were obtained from patients before their first surgery using a vacuum collection vessel (BD Vacutainer®SSTTM tube) containing separation gel. At 4℃, 1800g, centrifuge for 10 min, the serum sample was collected as supernatant and stored at -80℃ for long-term storage.

1-3 ml of peripheral blood was drawn from each patient and 100 μL of the sample was transferred to a centrifuge tube. 400 μL of methanol was added to the tube and vortexed for 60 s. After centrifugation at 12000 rpm for 10 min at 4 ℃, the supernatant was transferred to a 2 mL centrifuge tube, concentrated and dried under vacuum. The sample was reconstituted with 150 μL of 2-chlorophenylalanine (4 ppm) in 80% methanol, and the supernatant was filtered with a 0.22 μm membrane to obtain the sample for testing. 20 μL of each sample was mixed to prepare a quality-control sample, and the remaining samples were subjected to LC-MS analysis.

**2** Liquid Chromatography-Mass Spectrometry (LC-MS) method

LC-MS was performed using a Thermo Vanquish system with an autosampler temperature of 8 ℃, a flow rate of 0.25 mL/min, and a column temperature of 40 ℃. 2 μL of the sample solution was injected for gradient elution. The mobile phase for positive ion mode contained 0.1% formic acid in water (B1) and 0.1% formic acid in acetonitrile (A1), and the mobile phase for negative ion mode was comprised of 5 mM ammonium formate in water (B3) and acetonitrile (A3). The gradient elution program was as follows: 0–1 min, 2% A1/A3; 1–9 min, 2%–50% A1/A3; 9–12 min, 50%–98% A1/A3; 12–13.5 min, 98% A1/A3; 13.5–14 min, 98%–2% A1/A3; 14–20 min, 2% A1– positive mode; and 14–17 min, 2% A3–negative mode.

MS conditions were as follows: Thermo Q Exactive Plus mass spectrometer, electrospray ion source, positive and negative ionization mode, positive ion spray voltage of 3.50 kV, negative ion voltage of 2.50 kV, sheath gas of 30 arb, and auxiliary gas of 10 arb. The capillary temperature was set at 325°C. A full scan was performed with a resolution of 70,000, and the scan range was 81–1000. HCD was used for secondary decomposition, and the collision voltage was 30 eV. Unnecessary MS/MS information was removed using dynamic exclusion.

**3 LC-MC analysis**

3.1 Metabolic profiling Chromatographic peaks were selected respectively in positive and negative ion modes to investigate the stability of their retention time and peak area in the Non-RAIR and RAIR patients. The retention time RSD and peak area RSD of chromatographic peaks in positive ion mode as well as the retention time RSD and peak area RSD of the peaks in negative ion mode were used as the basis for assessing the stability of the analytical system.

3.2 Multivariate statistical analyses The data were analyzed to reveal precursor molecules in positive and negative ion modes. Principal component analysis (PCA) was performed to generate new characteristic variables by linear combination of metabolite variables according to a certain weight. Each group of data were categorized based on the main new variables (principal components), and samples with poor repeatability (outlier samples) and abnormal samples were removed. The obtained PCA model reflects the original state of the metabolomic data, and the PCA method was used to respectively analyze the positive and negative ion modes.

3.3 Metabolic pathway analysis The identified differential metabolites were subjected to heat map analysis, ROC curve analysis, and metabolic pathway analysis using the metaboanalyst database (http://www.Metaboanalyst.ca).

Supplementary figures and tables


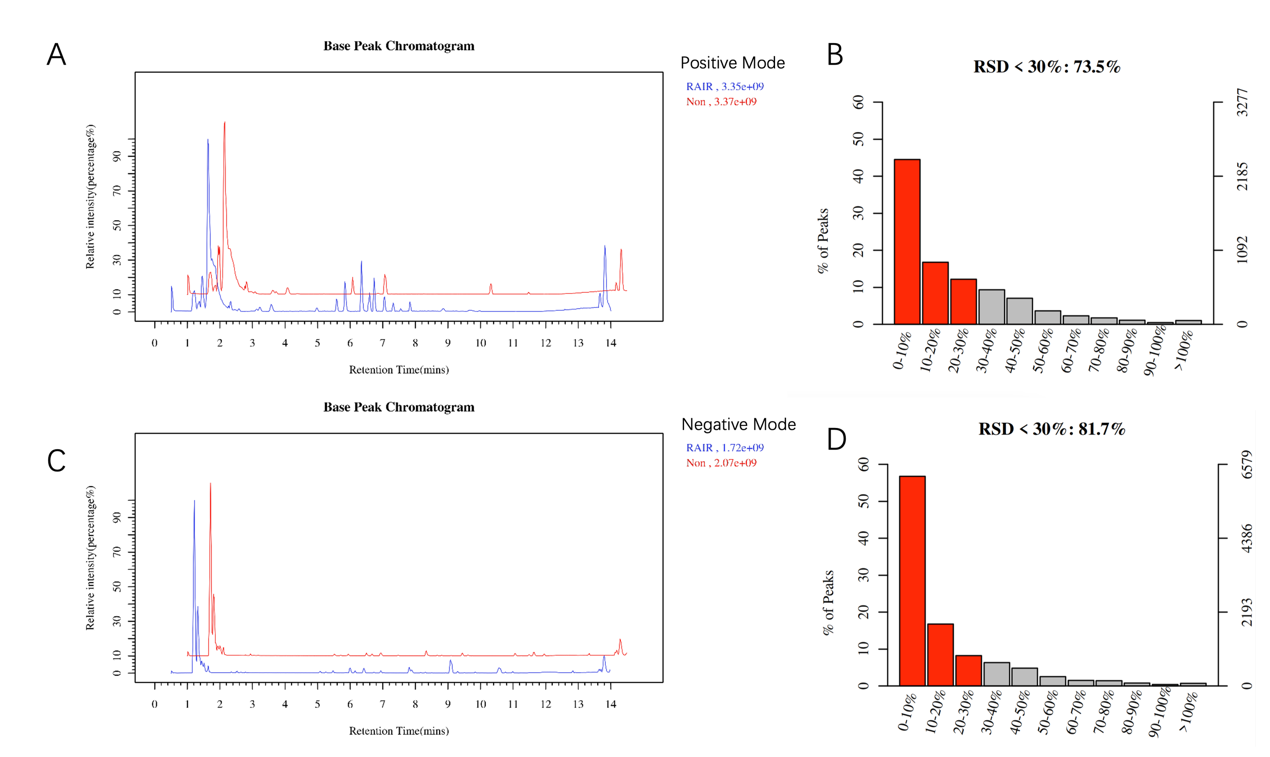


FigS1. Typical sample base peak chromatogram in positive and negative ion mode


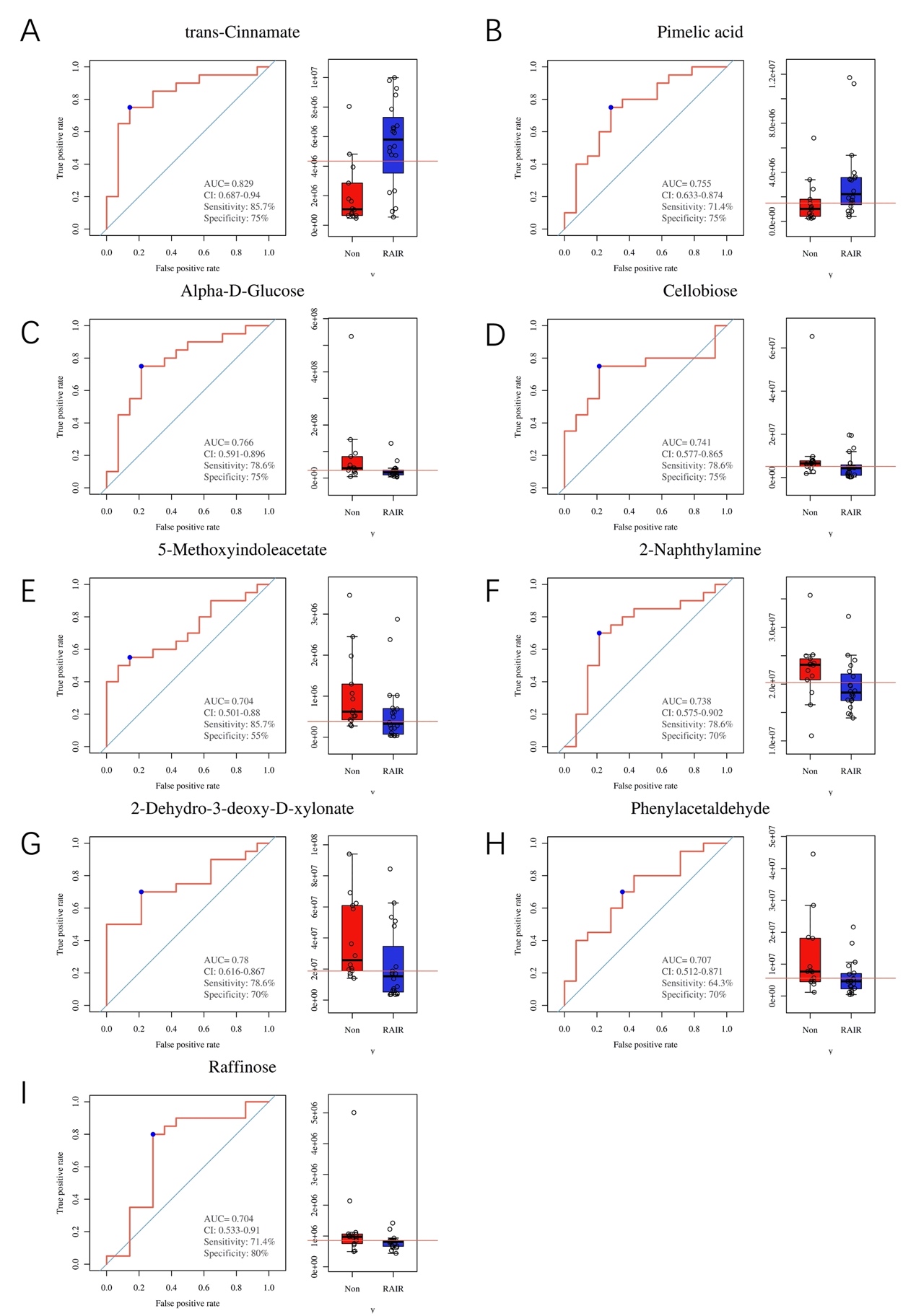


FigS2. Receiver operating characteristic (ROC) curves of other 9 Metabolic compounds and its change in RAIR and no-RAIR groups


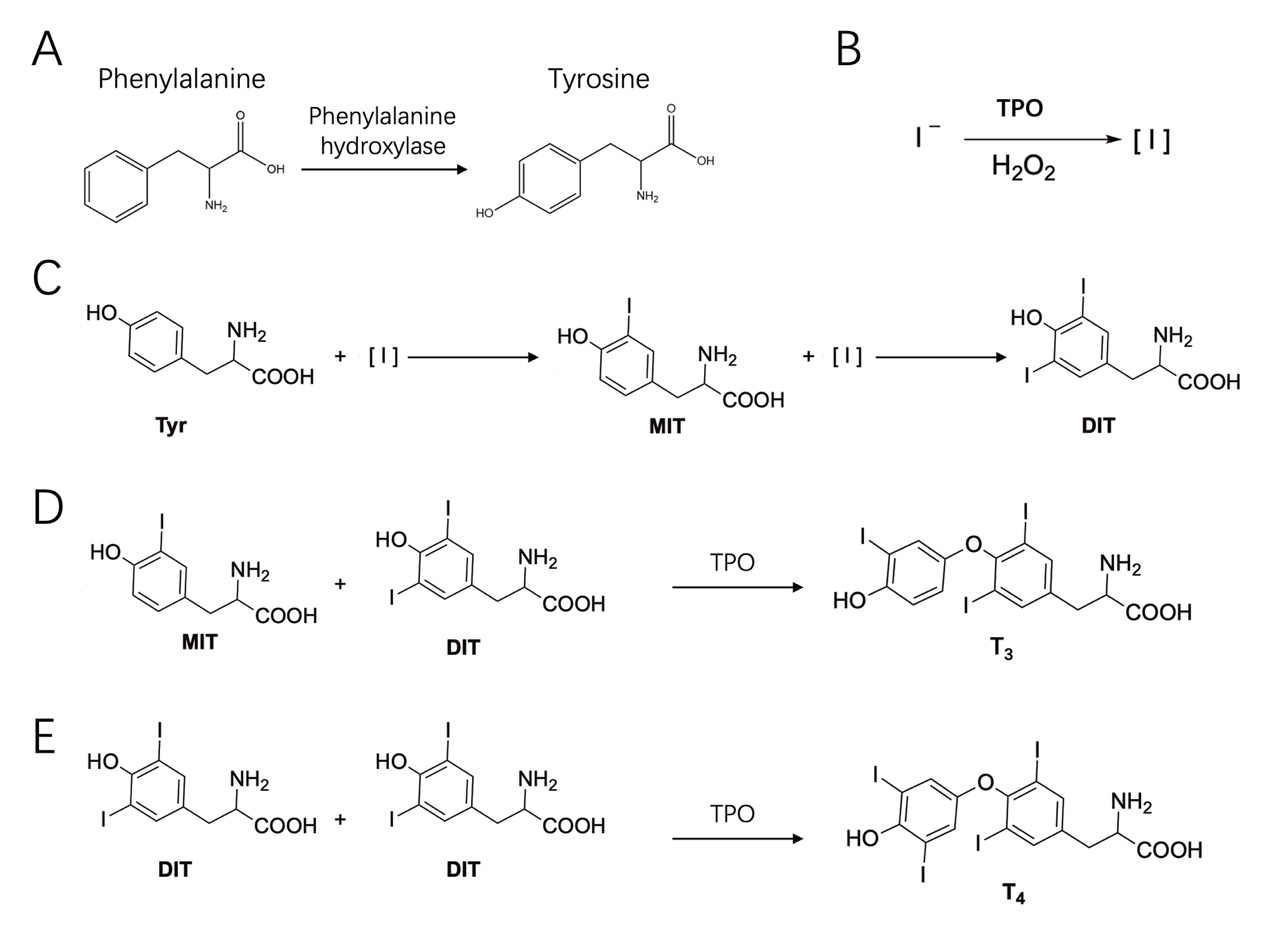


FigS3**.** The chemical reaction of phenylalanine with tyrosine and the iodination of tyrosine to produce the thyroid hormone. A, Conversion of phenylalanine into tyrosine by phenylalanine hydroxylase. B, Conversion of iodine ion to elemental iodine by thyroid peroxidase (TPO) and hydrogen dioxide. C, Iodization of tyrosine to mono-iodotyrosine (MIT) and di-iodotyrosine (DIT). D, Tri-iodothyronine (T_3_) is produced by the coupling of MIT and DIT by TPO. E, Production of tetra-iodothyroxine (T_4_) by the coupling of MIT and DIT by TPO.

Table S1. Tyrosine, MIT, and DIT are produced differently in different chemical systems

|  | Chemical Reaction System | | | | | | |
| --- | --- | --- | --- | --- | --- | --- | --- |
|  | | **I** | II | III | IV | V | VI |
| Tyrosine | | **Tyr** | Tyr | Tyr | Tyr | Tyr | Tyr |
| Iodine | | **I^-^** | I_2_ | I^-^/I_2_ | I_2_ | I_2_ | I^-^ |
| Hydrogen peroxide | | **H_2_O_2_** | H_2_O_2_ | H_2_O_2_ | - | - | - |
| Thyroid peroxidase | | **TPO** | TPO | TPO | TPO | - | - |
| Result | |  |  |  |  |  |  |
| Tyr | |  |  |  |  |  | NO |
| MIT^*^ | | **+++** | ++ | ++ | + | - | - |
| DIT^#^ | | **+++** | ++ | ++ | + | - | - |

* MIT Counts(10^4), ≤5（+）；5~10（++）；≥10（+++）

#DIT Counts(10^4), ≤10（+）；10~20（++）；≥20（+++）
